# Targeting Carbonic Anhydrase IX Activity and Expression

**DOI:** 10.3390/molecules20022323

**Published:** 2015-01-30

**Authors:** Brian P. Mahon, Melissa A. Pinard, Robert McKenna

**Affiliations:** Department of Biochemistry and Molecular Biology, College of Medicine, University of Florida, Gainesville, FL 32611, USA

**Keywords:** carbonic anhydrase IX, tumor hypoxia, prodrug, sulfonamide, RNAi-technology, cancer

## Abstract

Metastatic tumors are often hypoxic exhibiting a decrease in extracellular pH (~6.5) due to a metabolic transition described by the Warburg Effect. This shift in tumor cell metabolism alters the tumor milieu inducing tumor cell proliferation, angiogenesis, cell motility, invasiveness, and often resistance to common anti-cancer treatments; hence hindering treatment of aggressive cancers. As a result, tumors exhibiting this phenotype are directly associated with poor prognosis and decreased survival rates in cancer patients. A key component to this tumor microenvironment is carbonic anhydrase IX (CA IX). Knockdown of CA IX expression or inhibition of its activity has been shown to reduce primary tumor growth, tumor proliferation, and also decrease tumor resistance to conventional anti-cancer therapies. As such several approaches have been taken to target CA IX in tumors via small-molecule, anti-body, and RNAi delivery systems. Here we will review recent developments that have exploited these approaches and provide our thoughts for future directions of CA IX targeting for the treatment of cancer.

## 1. Introduction

Hypoxia is a condition commonly seen in metastatic tumors where cells are deprived of oxygen due to rapid proliferation and a shift in their metabolism [1]. Specifically, hypoxic tumor cells outgrow their blood supply leading to regions of low oxygen concentration (typically ≤1% of overall oxygen content) as well as a decrease in extracellular pH (~pH 6.5) in the tumor microenvironment [1,2]. This hypoxic stress induces a shift in the tumor cells general metabolism from oxidative phosphorylation in the mitochondria to aerobic glycolysis in the cytosol as the main energy source. Interestingly, this metabolic shift remains present in the tumor cells regardless of the amount of the available O_2_ in the given environment; a phenomenon often described as the Warburg effect [3]. Since these tumor cells rapidly use glycolysis, increased amounts of lactic acid are exported from the cell, thus lowering the extracellular pH. As a result, there is an upregulation of pH homeostasis factors in tumor cells to establish a regulated intracellular/extracellular pH gradient [3,4].

Since the 1930s it has been well established that there is a correlation between tumor hypoxia and a resistance to radiation therapy [5,6]. In addition, hypoxic tumors have shown to also present a resistance to common chemotherapeutics and a high probability of metastases; hence tumor hypoxia has been associated with a poor patient prognosis [7]. Hypoxia inducible factors (HIF) are key regulators of the hypoxic-induced stress response in both normal and tumor cells. Specifically, increased HIF-1 has been associated with activating hypoxia-inducible genes that express hypoxia-responsive elements (HRE) that upregulate elements associated with metabolism, cell proliferation, drug resistance, pH regulation, angiogenesis, metastasis, and the overall progression of cancer [8]. In order to survive in the acidic microenvironment these tumor cells must be able to maintain an intracellular pH at or near physiological levels (pH 7.4) [9]. Therefore carbonic anhydrase (CA) activity is key in this regulatory process.

CAs are a family of zinc metalloenzymes that catalyze the reversible hydration of carbon dioxide to bicarbonate and a proton [10]. Humans express 15 CA isoforms (alpha-class CAs), and of these CA IX and CA XII have been shown to be associated with tumors. Both these enzymes are transmembrane isoforms with an extracellular catalytic domain, and show high expression in solid tumors while exhibiting low expression in normal tissues (CA IX expression only) [11]. CA IX however, has been shown to be more prevalent in solid tumors compared to CA XII, and as a result has been marked as a therapeutic target for aggressive cancers [7,12,13].

CA IX expression directly correlates to an upregulation of HIF elements, and has been shown to play a role in tumor cell survival, proliferation, migration, growth, adhesion, pH regulation, and cell-signaling pathways [14,15,16]. The minimal expression of CA IX in normal tissues and its location on the external interface of tumor cells have made it an attractive therapeutic target. As a result, several methods have been employed to try to target CA IX in terms of isoform selective small-molecule inhibition, location specific targeting, knockdown using RNAi technology, and more recently antigenic targeting of CA IX as a means to deliver anti-cancer therapeutics directly to tumor.

In this review we will present a brief overview of the biochemical and biophysical properties of CA IX, discuss current technologies used to target the enzyme for the treatment of several cancers, and speculate on novel methods of delivering therapeutic applications.

## 2. CA IX Structure and Function

**Figure 1:**
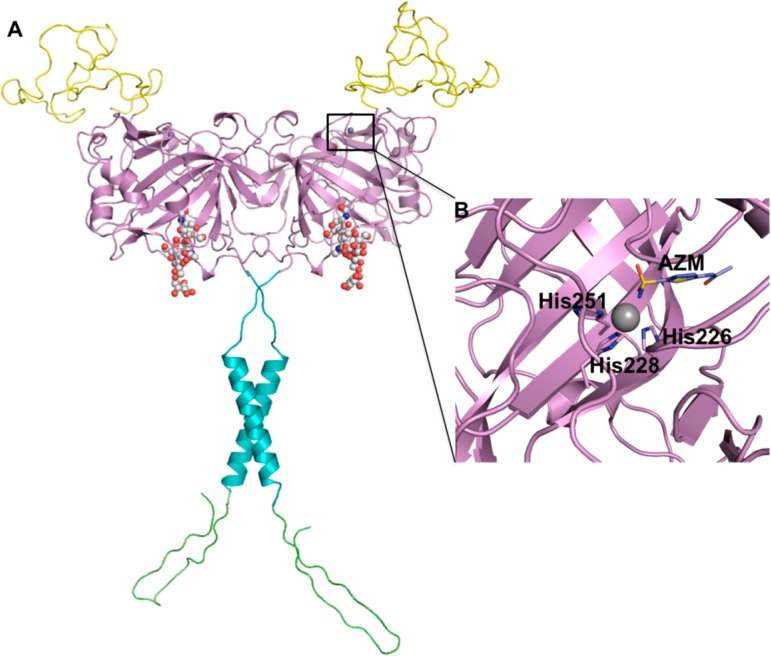
(**A**) Schematic diagram of CA IX structure. Proteoglycan-like domain (PG, yellow), catalytic domain (CA, violet), a transmembrane anchor (TM, cyan), and an intracellular domain (IC, green). Spheres represent glycosylation sites. The PG domain was generated using a structure prediction server *Robetta* [24] and the CA domain is from the coordinates of the CA IX crystal structure (PDB ID: 3IAI). The TM anchor and IC domain were generated using *Chimera* [25] and *COOT* [26] software packages, respectively. This figure was adapted from: Mahon *et al.* [27] (**B**) Acetazolamide (AZM) bound in the active site of CA IX (PDB ID: 3IAI). Figure was created using *PyMol* [28].

**Figure 2:**
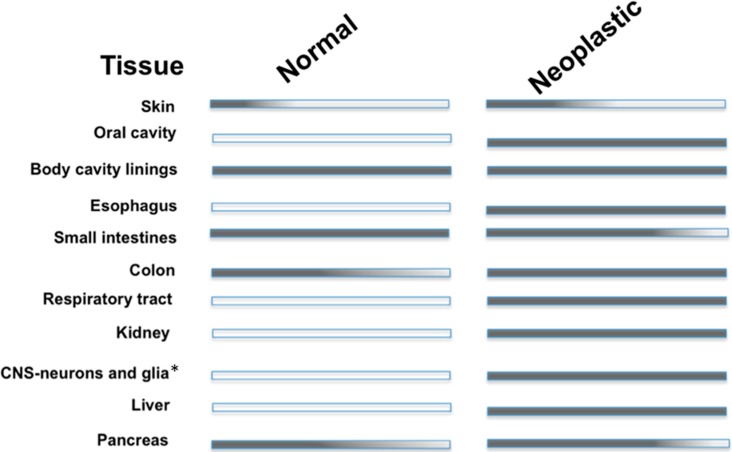

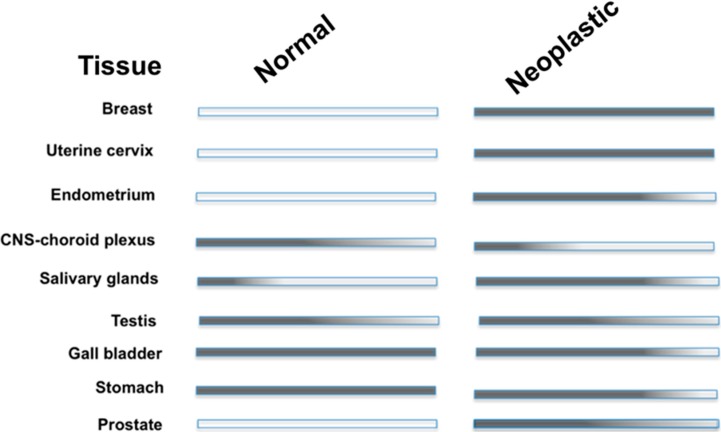
CA IX expression in adult human tissue. Note that (*) indicates high-grade tumor tissues.

The *CA9* gene encodes for a 459 amino acid transmembrane glycoprotein that exists as a homodimer. It is comprised of: a proteoglycan-like domain (PG) (59 aa), catalytic domain (CA) (257 aa), a signal peptide domain (which is removed prior to enzyme maturation) (37 aa), transmembrane domain (TM) (20 aa), and a C-terminal intracellular domain (25 aa) [17] (Figure 1A). Mass spectroscopy and X-ray crystallography have confirmed the presence of an intermolecular disulfide bridge between adjacent Cys137 residues of the mature homodimer that, coupled with a region of hydrophobic residues, are proposed to stabilize the dimer interface [18,19]. *N*-linked and *O*-linked glycosylation sites also exist at Asn 309 and Thr 78, respectively [20]. The catalytic domain of CA IX is structurally homologous to the alpha-CAs with high amino acid conservation within the active site [21]. In CA IX three histidine residues (His 226, 228 and 251, as numbered in the full length aa sequence) coordinate the zinc ion at the base of the active site cleft; in the crystal structure (PDB ID: 3IAI) acetazolamide (AZM) displaces a zinc bound water/hydroxide (Zn-OH/H_2_O) molecule maintaining a tetrahedral coordination about the zinc ion (Figure 1B). Variability between the CA isoforms occurs in the hydrophobic and hydrophilic pockets of the active site and surface amino acids [19,20,22] (Figure 2). The structural and amino acid conservation that exists between the active sites of human CA isoforms has made it difficult to design CA IX specific inhibitors and avoid off-target inhibition of other CAs that are ubiquitously expressed in normal tissue [23].

The catalytic efficiency of CA IX is fast and comparable to that of CA II; CA II exhibits a *k_cat_* of 1.4 × 10^6^ while CA IX has a *k_cat_* of 3.8 × 10^5^ [29,30]. The presence of the PG domain in CA IX is unique compared to the other CA isoforms and is thought to be responsible for its cell adhesion capability and maintaining its catalytic activity in the acidic tumor microenvironment [27].

CA IX’s most critical role is thought to be extracellular pH regulation, especially in the tumor microenvironment. Proliferating cancer cells often produce large amounts of lactate, carbon dioxide and protons during oncogenic metabolism making CA function pivotal in tumor cell survival. These metabolic end products accumulate in the extracellular environment and significantly lower the extracellular pH. In order to maintain a near physiological intracellular pH, bicarbonate anions generated by CA IX during the hydrolysis of carbon dioxide are transported into the cell via anion transporters to buffer intracellular pH levels. In addition protons produced from the reaction remain extracellular thus contributing to the acidic nature of the tumor milieu [31]. Disruption of this regulatory pathway would therefore have detrimental effects on overall tumor cell survival.

## 3. HIF-1 Regulates CA IX Expression

HIFs are major regulators of stress induced responses in tumor cells and CA IX expression has been observed to be directly linked to an upregulation of HIF-1 [5]. HIF-1 is a heterodimeric complex, consisting of an α- and β-subunit (HIF-α and HIF-β, respectively). The HIF-α subunit exists as three isomers: 1, 2 and 3. During activation of hypoxia-inducible genes via HIF mediated pathways, the HIFα-β heterodimeric complex forms in the cytosol and is trafficked to the nucleus [32,33]. Formation of this heterodimer is the rate-determining step of in the expression of HREs since in non-hypoxic stress induced conditions the α-subunit is quickly degraded via the Von Hippel-Linadau (VHL) regulatory pathway [9,32,33,34]. HIF-α and HIF-β are ubiquitously expressed in both normal and neoplastic tissue [35]. Activation of HIF-1 is mediated by several factors including changes in overall O_2_ content, an up regulation of inflammatory factors, activation of several signaling pathways, and in the case of renal cell carcinoma (RCC) it is induced by VHL dysfunction [35,36,37]. HIF-1 trafficking to the nucleus causes the activation of several hundreds of genes, which either directly or indirectly play a role in tumor cell migration and survival [38,39,40]. One of these HREs is the gene expressing CA IX.

## 4. CA IX Expression in Normal *vs.* Neoplastic Tissue

In a non-disease state CA IX expression is limited to the gut epithelium; specifically, the basolateral surfaces of the cryptic enterocytes of the duodenum, jejunum and ileum [41]. The most prominent levels of CA IX are seen in these proliferating crypt cells suggesting CA IX may be involved in intestinal stem cell proliferation and regulation of certain metabolic functions [42].

Northern blot and immunohistochemical staining have also confirmed CA IX expression in the ovarian coelomic epithelium, cells of hair follicles, pancreatic ductal cells and fetal rete testis [43,44]. In addition high levels of CA IX are observed in developing embryonic tissues of the gut, lung and skeletal muscle and decrease in adult tissues [43]. These observations indicate CA IX expression is primarily associated with areas of low pH and high rates of cell proliferation in normal tissues. Whether or not this makes CA IX a regulatory element in normal tissues has not been confirmed.

CA IX is ectopically expressed in a variety of neoplastic tissues. Expression has been observed in malignancies of the breast, lung, kidney, colon/rectum, cervix uteri, oral cavity (head/neck), gallbladder, liver, brain (high-grade), pancreas, and gastric epithelium [31,43,44]. A list of the differential expression patterns of CA IX in normal and neoplastic tissue is presented in Figure 2. No differences exist between the cDNA of CA IX isolated from normal and tumor tissues, which implies similar physiological function in both tissues. As mentioned previously, CA IX expression depends on HIF-1 activation (via the upregulation of HIF-1α or the down regulation of VHL). Specifically, the activation of the HIF-1 mediated pathway that induces CA IX expression can be due to a reduction in cellular O_2_ levels, an activation of signaling pathways via the presents of growth factors and inflammatory response elements, and in some cases due to mutations in the tumor suppressor, VHL as seen in cases of renal cell carcinoma (RCC) where CA IX is homogenously expressed [27]. More recently, CA IX has shown to have significant expression levels in stromal cells that are engaged in a molecular cross-talk circuitry with cancer cells. Specifically, CA IX has been shown to be expressed in cancer-associated fibroblasts (CAFs) via redox-based stabilization of HIF-1. It is postulated that expression of CA IX in CAFs provides the acidic extracellular environment necessary to drive epithelial-mesenchymal transitions (EMTs) in adjacent cancer cells [45]. Summation of these findings indicates CA IX as a diagnostic marker of events of tumor hypoxia in many solid tumors [43].

CA IX expression levels also serve as prognostic markers for several cancer types. Specifically, patients suffering from brain, breast, cervical, rectal or lung cancer that also display high levels of CA IX typically show a poorer prognosis. In contrast, for clear cell renal cell carcinoma (CCRCC) patients low CA IX levels indicate poor clinical outcome [16,27,31]. CA IX’s contribution to maintaining the hypoxic tumor microenvironment is highly correlated to patient prognosis thus making it both a biomarker and drug target [27].

## 5. Structural Homology among Human CAs

The 15 human CA isoforms expressed are CA I, II, III, VA, VB, VI, VII, VIII, IX, X, XI, XII, XIII, XIV, and XV, of which 12 display catalytic activity. CA VIII, X and XI are non-catalytic and are termed CA related proteins (CA-RPs) [10]. Apart from differences in catalytic efficiency among the active isoforms they also differ in cellular distribution and localization, and as a result are involved in various physiological processes [10,18,29]. Furthermore, aberrant expression of the enzymes is commonly associated with a host of diseases. These include: glaucoma (CA II, IV), cancer (CA IX, XII), edema (CA II), sterility (CA XIII), altitude sickness (CA II), obesity (CA VA) and hemolytic anemia (CA I) [29,46].

**Figure 3:**
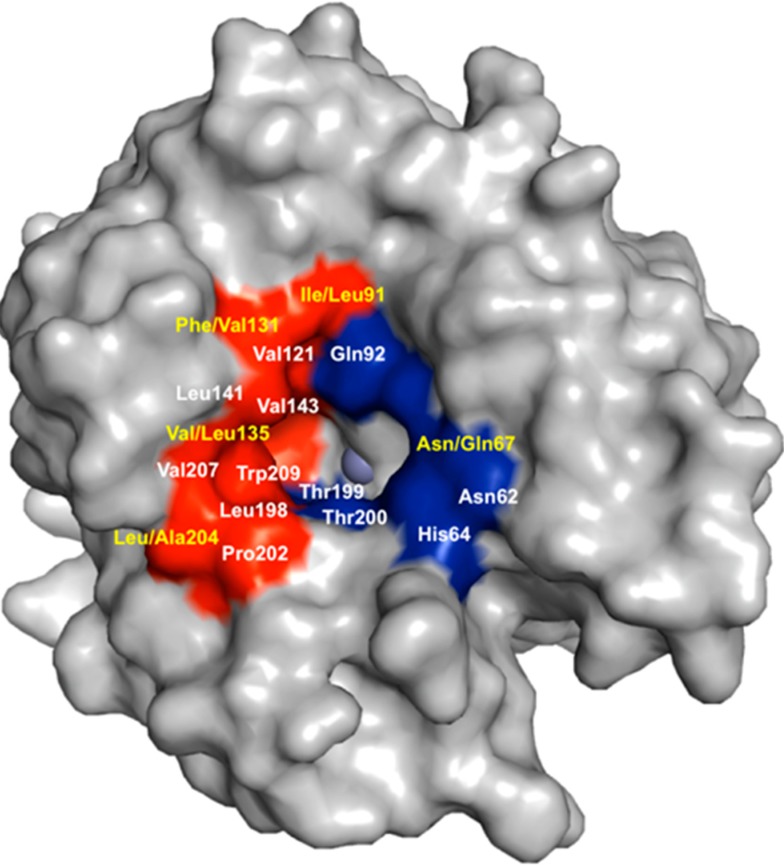
Surface rendition of CA IX (PDB ID: 3IAI). Residues in the hydrophobic (red) and hydrophilic (blue) cleft are as labeled; residues conserved (white) and those that differ (yellow) between CA II and CA IX. Figure made using *PyMol* [28].

The alpha-class of CAs are highly homologous inferring that they also have overlapping functional roles. The human CAs share at least 30% primary sequence identity among themselves with major of conservation within the active sites of all the catalytic isoforms [23]. The active site is located at the bottom of a conical cavity where a zinc ion is positioned in a tetrahedral coordination with three histidine residues and a water/hydroxide molecule [47]. The catalytic domain of CAs is also characterized by conserved hydrophobic and hydrophilic regions adjacent to the entrance to the active site. Figure 3 highlights the similarities and differences between CA II and CA IX in and around the active site. The high amino acid conservation existing among the human isoforms has made it difficult to design isoform specific CA inhibitors (CAIs) targeting CA IX that exhibit limited off-target effects [48].

## 6. Improving Classic CAIs

**Figure 4:**
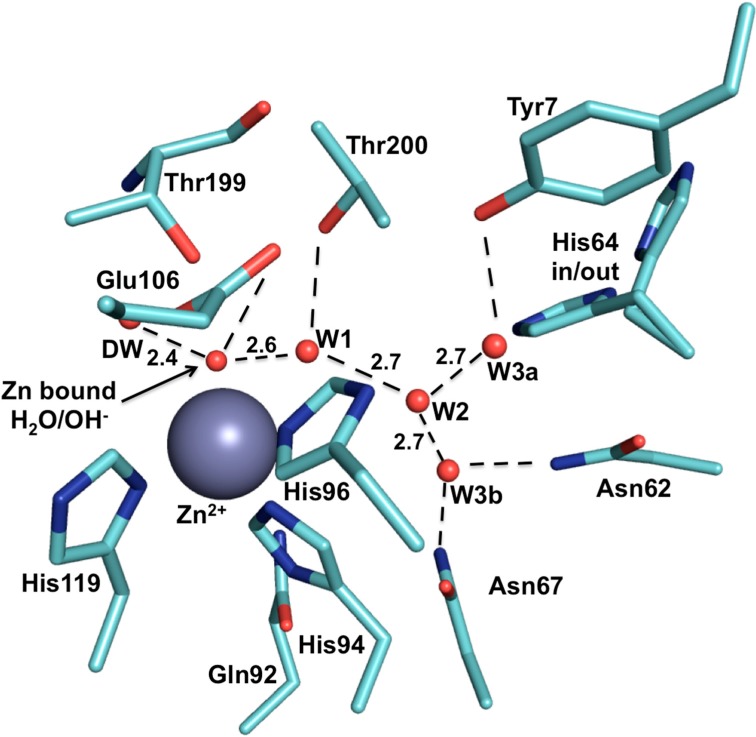
The active site of CA II (PDB ID: 3KS3). The zinc ion is represented by a grey sphere and is coordinated by His 94, 96 and 119, and a H_2_O/OH^−^ molecule. The zinc bound H_2_O/OH^−^ binds at a distance of 1.9 Å away from the zinc ion. Water (W) are represented by red spheres while hydrogen bonds (H-bonds, Å) are represented by dotted lines. This figure was made using *PyMol* [28].

CAIs have been extensively studied and their inhibition mechanisms are well established [49]. Sulfonamide-based compounds are the most potent and most utilized among the CAI classes. These compounds bind to the zinc ion via a sulfonamide as the zinc-binding group (ZBG) in a deprotonated form displacing the zinc bound water/hydroxide molecule while still maintaining the tetrahedral coordination about the zinc ion [29,49] (Figure 1B). Though some sulfonamides display inhibition constants in the sub-nanomolar range for CA IX, these also inhibit other CAs. This is due to the conserved architecture of the active site among the human CAs. For all the catalytic human CAs, the three histidines coordinating the zinc, Thr 199 (CA II numbering; termed the “gatekeeper”), and Glu 106 are conserved [21]. Both T199 and E106 play roles in catalysis [30,50]. T199 hydrogen bonds to the zinc bound water/hydroxide via its OH group, while E106 hydrogen bonds to T199 [51] (Figure 4).

Small molecular weight CAIs that utilize a ZBG tend to bind deep into the active site cavity and do not make extensive interactions with amino acids that vary between the CA isoforms, thus contributing to their indiscriminatory inhibition profiles. As a result, alternative approaches have been developed for better isoform specific CAIs, with the “tail-approach” being one of the most successful. In the “tail approach” a chemical moiety (known as the tail) is appended onto an organic scaffold of a ZBG (for example heterocyclic or aromatic) [29,49]. This tail elongates the inhibitor allowing it to make extensive interactions with amino acids towards the outside of the active site. The addition of these tails can also alter the properties of the CAI, for example making it more soluble by the addition of a tail that is hydrophilic in nature, or manipulating the overall charge of the compound; such as cationic CAIs. The use of structure-based drug design has proven a valuable technique to exploit the subtle differences existing between the active site of the various isoforms. For example, utilizing steroidal based sulfonamides as lead compounds has led to the development of several similar CAIs that are able to exploit CA IX’s larger hydrophobic pocket by increasing the number of hydrophobic interactions via van der Waals contacts [27].

Despite the promise of structural exploitation of the CA IX active site to improve upon current and novel CAIs, the expression and crystallization of wild type CA IX has been an arduous challenge and thus made it difficult to carry out extensive structural analysis. As such our group has engineered a CA IX-mimic, which is a modified CA II (an enzyme that is routinely expressed and crystallized) that contains active site mutations specific to CA IX. This has provided a useful template to rapidly analyze and predict modes of binding of CAIs to CA IX [52,53]. Structural analysis of several CAIs has made it possible to design drugs that exhibit both location specific targeting and “prodrug like” properties that have shown to be useful in selectively inhibiting CA IX. These approaches will be discussed in more detail in the following sections.

Apart from the development of small-molecule inhibitors, CA IX specific antibodies and their conjugates have also been engineered with some currently in Phase III clinical trials (RECENARX^®^). M75 and G250 are two such monoclonal antibodies that recognize the enzymes proteoglycan domain [54,55]. Upon binding to CA IX these antibodies cause a reduction in tumor cell adhesion and motility, and induce natural killer cells to target tumor cells for eradication [20,25,56]. The development of monoclonal antibodies with high binding affinity eliminates the problem of off-target effects commonly encountered in CAI drug design [57]. Both antibodies have been extensively reviewed (see reference [7,20,57]) and therefore we will not discuss these therapies in detail. However in this review we will expand on utilization of such antibodies as delivery mechanisms targeted cancer therapeutics.

## 7. Location Specific Small-Molecule Inhibitors Target CA IX

**Figure 5:**
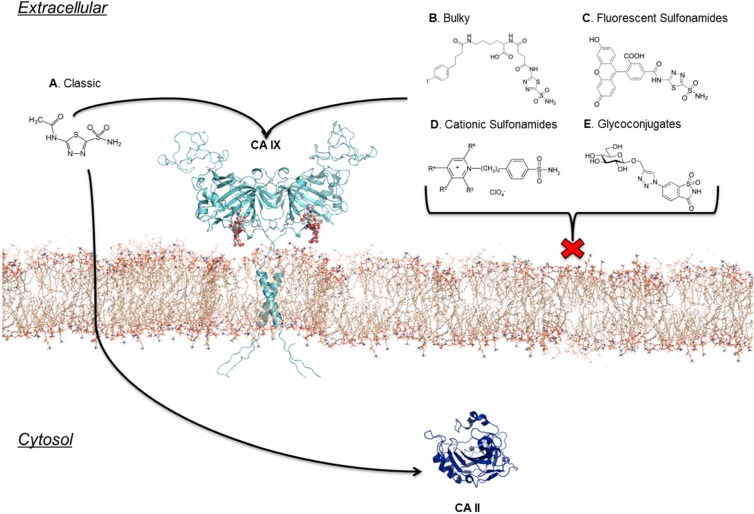
CAIs designed to target the extracellular CA domain of CA IX as compared to classic inhibitors. (**A**) Classic (**B**) Bulky (**C**) Fluorescent (**D**) Cationic sulfonamides (**E**) Glycoconjugates all show extensive differences in terms of CA IX specific targeting potential.(Figure was adapted from [67] and made using *PyMol* [28] and *ChemDraw* [68] software packages).

The extracellular location of the active site of CA IX presents an alternative method of targeting the enzyme in tumor cells [20,23,25]. Specifically, CAIs can be designed that have physiochemical properties that allow them to be impermeable to the plasma membrane; hence decreasing the chance of inhibiting off-target cytosolic CAs observed by classic CAIs [23] (Figure 5A). This presents a drug design strategy that incorporates location specific targeting of CA IX rather than exploiting differences in inhibition profiles alone. To date several compounds that show limited membrane permeability have been synthesized and designed. Such compounds utilize bulky chemical moieties, such as in albumin-acetazolamide, or exploit charged moieties in the form of fluorescently labeled sulfonamides or cationic sulfonamide derivatives [7,58,59,60,61] (Figure 5B–D, respectively). The design of such CAIs employ essentially two distinct rationales: (1) high molecular weight compounds that are simply too bulky to cross the plasma membrane, or (2) a cationic moiety that is incapable of permeating into the reduced cytosolic environment [62,63]. Despite both types of compounds showing favorable inhibition and membrane impermeability, the use of cationic sulfonamides has shown to be the more feasible option for drug development since high molecular weight compounds often induce potent allergic reactions and reduced bioavailability *in vivo* [60,64,65]. As a result several cationic sulfonamides have been developed using quaternary ammonium sulfate (QAS) as a lead compound, or fluorescently labeled sulfonamide derivatives [7,23,58,59,60,61,64,66].

Glycoconjugated sulfonamides, a more recent class of CAIs, have shown to exhibit both membrane impermeability and isoform selective inhibition of CA IX [52,53,69,70,71,72] (Figure 5E). These particular CAIs utilize benzene sulfonamides, sulfonamides, or cyclic secondary sulfonamides conjugated to a mono- or disaccharide tail [66,69,73]. The design of these CAIs was through the influence of the clinically used Topiramate (anti-epileptic therapeutic) [66,69,73]. Most likely the reason these compounds do not permeate into the cell is due to their high molecular weights, and the addition of a sugar moiety that is not easily transported [69,70,71]. Furthermore, unlike previously used bulky sulfonamide derivatives, the addition of a sugar moiety allows these CAIs to maintain water-solubility, and thus maintain good bioavailability [69,70,73]. Another promising aspect is that these CAIs show an impressive inhibition profile, with a >1000-fold selectivity for CA IX over CA II in some cases [52,53,70]. Also, the carbohydrate attachment presents an area of manipulation on these CAIs where cleavable ester bonds can be added to the carbonyls of the carbohydrate tail allowing the CAI to be “packaged” in the form of a prodrug [70]. Although these compounds present great promise in terms of developing a drug for CA IX, the use of carbohydrate moieties poses a potential dilemma. That is, the use of a carbohydrate, specifically a monosaccharide, might unintentionally interact with glucose transporters, similar to statins, in which myotoxicity was observed [72,74]. However, this notion has not been tested. Interestingly, a way to circumvent such an issue would be the development of sucrose-based conjugates that would have no interactions with specific transporters due to the lack of sucrose transporters in human tissue [71]. Interestingly, the current disaccharide-conjugates that have been developed into CAIs utilize a galactose moiety and show stronger inhibition for CA II *versus* CA IX [69]. Although these compounds will not enter the cytosol, they may not bind to CA IX tightly enough to be considered a valid drug candidate. However, utilization of other disaccharide-based compounds, such as the suggested sucrose-conjugate mentioned previously, might show higher inhibition for CA IX, and thus present a CAI that is selective for CA IX in both location specificity and direct inhibition.

## 8. Taking Advantage of “Prodrug” Properties to Inhibit CA IX

Prodrugs represent ~20% of all approved drugs since the early 2000s [75]. As such various types of prodrugs, or potential prodrugs exist that target the tumor microenvironment in either an active or passive approach. Active Prodrugs rely on either the present of esterases, or in the case of anti-cancer drugs, the upregulation of extracellular matrix (ECM) proteases, such as matrix metalloproteinases (MMPs) or urokinase-type plasminogen activators (uPAs) (all are upregulated by HIFs), to become unmasked [76]. For instance doxorubicin, a common chemotherapeutic agent, can be conjugated with a peptide specific substrate or antigen recognized by the aforementioned ECM proteases and unmask when reaching its tumor targets [76,77]. This provides a tissue-specific approach to active prodrug delivered chemotherapies and reduce off-target effects.

Alternatively, passive prodrugs are unmasked when introduced to the hypoxic tumor microenvironment. As mentioned previously, the hypoxic tumor microenvironment has both reduced pH and O_2_. These microenvironmental conditions unmask passive prodrugs by (1) protonation by the slightly acidic pH, or (2) reduction via the absence of adequate O_2_ in the environment [76]. For example, utilization of ligands with coordination metal complexes that are redox capable have been implemented as prodrugs to target tumor microenvironments. Straplatin, an oxidized analog of the common chemotherapeutic cisplatin, is synthesized with a less biologically reactive Pt(IV) coordination center, and when introduced to the reduced tumor microenvironment Pt(IV) reduces to Pt(II) causing an interaction with adjacent tumor cells by the same mechanism as cisplatin [76]. Similarly imidazotetrazine prodrugs have shown to be clinical useful as adjuvant cancer therapies coupled with radiation treatment [78]. These compounds take advantage of the slightly acidic microenvironment via a complex mechanism of action. They are first processed at physiological or slight alkaline pH forming intermediates. Once these intermediates have formed they can be protonated by the acidic tumor microenvironment causing an unmasking of the compound that allows for direct interaction with methylated DNA initiating a tumor cell killing response [78].

**Figure 6:**
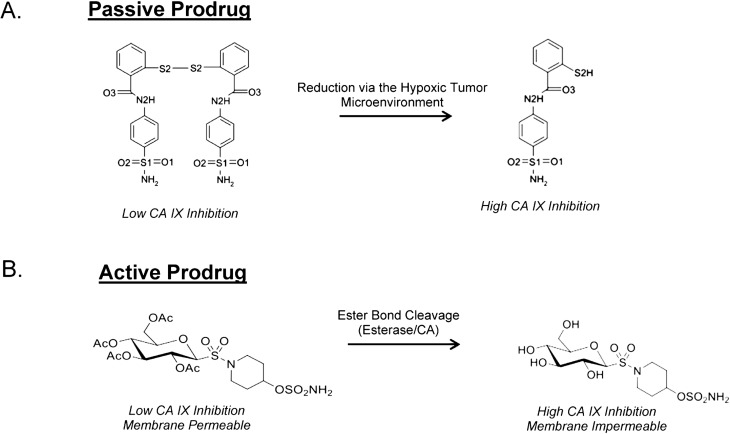
Prodrug CAIs. (**A**) Passive prodrug CAIs (4-(2-mercaptophenylcarboxamido) benzenesulfonamide) utilizes the microenvironmental changes to become unmasked. (**B**) Active prodrug CAIs require the presence of esterase activity either from ubiquitous esterases or the weak esterase activity of CAs to become unmasked.

CAIs that exhibit “prodrug-like” properties exists in the form of fluorescently-labeled sulfonamides, coumarin derivatives, glycoconjugates, and even photo-triggered compounds [7,59,60,62,71,79,80,81]. Not surprisingly, the first prodrug CAIs developed were not specifically designed to target CA IX. Instead most prodrug CAIs were designed to target CA II for treatment of glaucoma [81,82,83,84]. One of the most interesting of these compounds utilizes a water-soluble photolabile cage that masks a hydrophobic sulfonamide compound that is a potent inhibitor of CA II [82]. In its unmasked form, the compound is a very poor CA II inhibitor. Once delivered however, the compound can be unmasked after photoexposure and becomes a potent CA II inhibitor [78]. The first prodrug CAIs designed to target CA IX were developed by De Simone, *et al.* [85]. These compounds act as passive prodrugs via unmasking by the hypoxic niche. These CAIs utilize a sulfonamide derivative containing a reducible chemical moiety of dithiodi-aliphatic/aromatic acyl halides [86]. The masked forms of these CAIs contain a disulfide-linked bond that can be reduced by the tumor microenvironment to form thiol derivatives that are potent CAIs (Figure 6A). Interestingly, along with being reduced chemically by the tumor microenvironment studies have shown they can also be reduced enzymatically by thioredoxin-1 [85]. The most promising compound of this type, 4-(2-mercaptophenylcarboxamido) benzenesulfonamide, shows close to µM affinity for CA IX in its inactive state, and when unmasked the affinity increases ~60-fold for CA IX [85]. However there have been no studies that speculate on the membrane permeability of these compounds; hence they may still produce off-target inhibition *in vivo*. Similar CAIs include fluorescently labeled sulfonamides that were original designed to track CA IX expression in tumor cells. Serendipitously it was shown these compounds act as passive prodrugs by showing high affinity for CA IX once they enter the hypoxic tumor microenvironment [59,61]. In addition (as mentioned previously) these CAIs are membrane impermeable [7,59,62]. Carbamoylphosphonates are another class of CAIs that act as passive prodrugs and show good bioavailability. These CAIs utilize the slightly acidic tumor microenvironment for unmasking. In neutral pH these CAIs remain readily permeable to the plasma membrane. Once entering the acidic tumor milieu they become ionized and are membrane impermeable [70]. In addition, once ionized Carbamoylphosphonates show increased CA IX inhibition [70,87].

Active prodrug CAIs rely on either ubiquitously expressed esterases or the weak esterase activity exhibited by CA to be unmasked [63,75,76,80,88]. Such compounds include coumarins and thiocoumarins. These compounds undergo hydrolysis via the Zn-OH^−^ to form a 2-hydroxycinnamic acid product that binds irreversibly to the entrance of the active site of CA IX [7,59,60,80,87]. Unlike sulfa-based CAIs these compounds, in their hydrolyzed form, do not interact with the catalytic zinc. More interestingly, these compounds exhibit the highest isoform selective CA inhibition profile compared to all CAIs, such that the hydrolyzed product interacts directly with CA IX specific residues found in the hydrophobic and hydrophilic pockets [59,79]. Alternatively, the glyococonjugate compounds mentioned previously can as act as prodrugs (Figure 6B). These CAIs also rely on esterase activity to exhibit their CA inhibitory properties [76]. The general construct of these inhibitors is (1) a sugar moiety at the tail region of the compound, (2) a 1,2,3-triazole moiety, and (3) a benzene sulfonamide group to act as the primary ZBG [70]. The carbohydrate moiety acts as the site of prodrug masking/unmasking where acyl groups occupy carbonyls of the sugar forming cleavable ester bonds. The addition of acyl groups allows the compound to be readily transported across the plasma membrane and exhibit poor CA inhibition. Once these ester bonds are cleaved the compound will exhibit the opposing characteristics [69,70]. So far, two sugar moieties have been utilized for these CAIs; glucose and galactose. Interestingly, compounds containing a glucose moiety not only show the highest membrane impermeability when unmasked but also exhibit a higher affinity for CA IX over other CAs [70]. The galactose-conjugated compounds however do not show a good inhibitory profile for CA IX over other CAs, and in some cases were better inhibitors of CA II [70]. In addition, these compounds are water-soluble which has the potential to increase their bioavailability, however *in vivo* studies are still necessary to conclude these remarks [69,70]. Despite the lack of *in vivo* data available for such compounds, so far their potential as drug candidates remains very hopeful.

## 9. Using CA IX as a Cell-Surface Receptor to Deliver Anti-Cancer Therapeutics

In addition to targeting CA IX directly for anti-cancer treatments, alternative routes utilize the antigenic properties of the enzyme as a means to deliver therapeutic payloads directly to the tumor site. This is possible due to both the cell-surface location of CA IX and its over expression in various tumor types [89,90]. This makes delivery of antibodies or small-molecule inhibitors favorable. As discussed these attributes have already been utilized in the form of antibody targeting [54] and non-permeable small-molecule inhibitors [60,64,73] in order to target CA IX directly to induce an anti-tumorigenic response. However coupling a CA IX-specific targeting mechanism with a routinely administered anti-cancer therapy creates a tumor-specific delivery system that will allow for localized treatment of cancerous tissues. One such mechanism uses what is referred to as CA IX-directed immunoliposomes [89,91]. Liposomes have been commonly used for drug delivery, especially in oncology due their good biocompatibility, low toxicity, and low immunogenicity [89,92]. CA IX-directed immunoliposomes utilize a CA IX-specific antibody conjugated to a liposome carrier of an anti-cancer therapeutic that, upon binding of the antibody region to CA IX, can be taken up by the tumor cell. Currently this technology has been used to deliver liposomes carrying submicron magnetic particles to renal cell carcinoma tissue for use in hyperthermal treatments [91], and more recently to deliver liposomes carrying docetaxel (a commonly used chemotherapeutic) to be delivered to lung carcinoma cells [89]. In both cases a CA IX-directed immunolipsome system was able to facilitate tumor specific delivery of the therapeutic agent.

As an alternative of utilizing an antibody-based targeting system to deliver cytoxic payloads to tumor cells, low molecular weight ligands have been used for the same purpose [90,93]. Utilization of small-molecules to target CA IX for delivery of anti-cancer therapeutics has advantages over using antibodies, as they exhibit deeper tissue penetration, faster pharmacokinetics, lower immunogenicity, and they are able to be produced by total organic synthesis expediting their production [93]. Recently, the utility of this technology to target tumor cells has been shown using ligand conjugates consisting of either a bivalent small-molecule or a ligand-dye conjugate specific for CA IX linked to maytansinoid DM1 (a potent cytotoxic agent) via cleavable disulfide bonds [90,93]. In each case, CA IX-specific ligands conjugated to DM1 were able to specifically target tumor tissue and deliver the cytotoxic agent [90,93]. Results from these studies facilitate a use for combining the aforementioned strategies of CA IX selective drug development to applications of conventionally used anti-cancer therapies. Utilizing CA IX selective small-molecule inhibitors as delivery vectors for therapeutic agents could potentially provide novel approach to targeted cancer therapy.

## 10. RNAi Mediated Knockdown of CA IX as a Means to Abolish the Hypoxic Tumor Milieu

So far, we have discussed the potential ways to modulate CA IX activity via several small molecule CAIs. However, the extensive data concerning the physiological role of CA IX in solid tumors suggests that its primary function may go beyond pH regulation [7,27,62]. In addition it has been observed that physiological functions of CA IX vary between different types of primary tumors. This suggests that simply blocking CA activity in hypoxic tumors may not fully disrupt the tumor milieu in a way that can be detrimental to the overall tumors growth and survival. Therefore, complete eradication of CA IX expression in hypoxic tumors may be a more beneficial therapeutic tactic than targeting its activity only. A feasible way to therapeutically knockdown CA IX expression in tumors is through the use of RNA interference (RNAi) technology, which has already been implemented for the treatment of several diseases including cancer [94].

The mechanism of RNAi utilizes the endogenous miRNA pathway that regulates protein expression by posttranscriptional gene silencing [94,95]. The general pathway of miRNA (and also siRNA) biogenesis occurs via transcription by RNA polymerase II (RNA pol II) in the nucleus to form what is called the pri-miRNA, which exhibits a hairpin loop structure [96]. This pri-miRNA is further processed by RNase III Drosha into a ~60-80 nt-long precursor miRNA (pre-miRNA) that is also stabilized by a loop structure. The pre-miRNA can then be exported from the nucleus via Exportin 5 (Exp5). Once in the cytosol the pre-miRNA hairpin stem region is cleaved by Dicer to form a ~22 nt-long miRNA-miRNA duplex (or siRNA-siRNA) [94,96]. The duplex miRNA-miRNA is than incorporated into what is known as the RNA-induced silencing complex (RISC) which functions to degrade on of the miRNA duplex strands (known as the passenger strand) and further escorts and incorporates the remaining miRNA into its mRNA target sequence [94]. At this point events of translational repression, enhanced mRNA degradation, or site-specific mRNA cleavage can occur [94,96].

Efficient siRNAs are designed to completely integrate at specific locations on an mRNA target sequence by exact base complementation and induce complete mRNA degradation at that specific region [94]. A key difference between endogenous miRNA mediated silencing and utilization of short-hairpin (shRNA) constructs is that shRNA are typically incorporated downstream of the Drosha processing pathway, and without alteration can be exported to the cytosol and further processed by Dicer to form viable siRNA [94,96]. Both shRNA and pri-miRNA constructs have been designed and successfully delivered to target cells via viral-vector transduction or a liposomal delivery systems [94,97,98]. The advantage of packaging of siRNA in either a viral or liposomal delivery system is that it allows for increased longevity of the siRNA *in vivo*, and also allows for circumvention of problems associated with the innate immune response [94].

There are currently several therapeutic siRNAs in Phase I or II clinical trials, with a large percentage focused on targeting different forms of cancer [94]. Interestingly, none of these siRNA therapeutics utilize a viral-vector delivery system despite the advances made in the field of gene therapy. Although there are several viral-vectors that have shown therapeutic potential, one that has gained the most interest is adeno-associated virus (AAV), a single-stranded parvovirus [99]. AAV holds many promising attributes for gene delivery such as its ability to naturally infect primates, it is nonpathogenic, and its ability to be engineered in a recombinant form (rAAV) that lacks *rep* and *cap* genes making its delivered DNA almost completely episomal [94,99]. The packaging capacity of AAV is 4.7 kb, which is often regarded as a disadvantage depending on the gene target. However this is of no concern in terms of RNAi-based applications [100]. As such there have been several rAAV serotypes engineered that successfully transduce a wide range of tumors or cancer cells in preclinical studies [99,101,102,103,104,105,106,107]. Further, RNAi mediated technology has already shown its utility in preclinical studies involving treatments of blindness and Huntington’s disease among others [94,108,109,110]. This type of progress suggests that AAV-mediated delivery of siRNA may play a pivotal role in cancer therapies in the near future.

**Figure 7:**
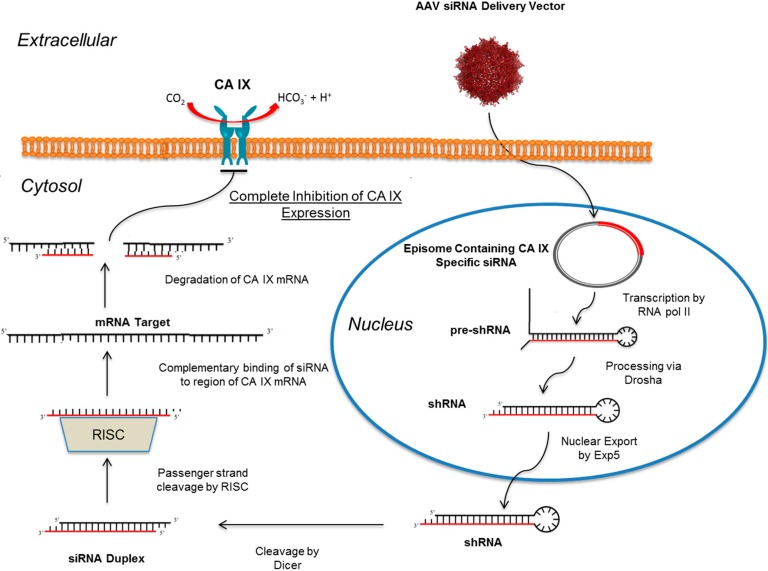
Pathway of AAV-mediated siRNA delivery to target CA expression in a tumor cell. The siRNA construct is delivered via a transduced AAV vector and its episome is trafficked to the nucleus. Within the nuclease the episomal DNA is transcribed by RNA pol II to produce a pre-shRNA construct that is further processed and transported to the cytosol where it becomes an siRNA duplex. The siRNA duplex is then processed by RISC and escorted to the specific mRNA region encoding for CA IX. Once bound, via complementary base-pairing, the CA IX mRNA is degraded and expression of CA IX is abolished. (This figure was adapted from [116] and AAV3B; PDB ID: 3KIC [117]).

Several studies involving modulation of CA IX expression in several types of cancer models utilize RNAi technology. For instance, specific siRNA technology has been used to modulate CA IX expression in liver, breast, kidney, brain, prostate and several other cancer types [20,44,111,112,113,114,115,116,117,118]. The successful knockdown of CA IX via siRNA mediation has shown to reduce primary tumor growth and proliferation in breast cancer models and also enhance effects of hexokinase inhibitors, promising compounds for treatment of liver cancer [112,113,115]. The few examples presented in this review suggests that combining RNAi technology to knockdown CA IX with a viral-vector delivery system may present a novel cancer therapy to inhibit tumor growth and proliferation. A model illustrating a proposed mechanism of AAV-delivered siRNA targeting CA IX expression is illustrated in Figure 7. This mode of therapy could act as an alternative to developing isoform selective CAIs in certain cancers where modulations in CA IX activity may not contribute greatly in disrupting tumor function (*i.e.*, liver cancers) [20,118]. However, much more research is needed to conclude the use of RNAi technology to knockdown CA IX via an AAV delivery system. Overall the plethora of data available in both fields can produce an exciting new method for treating aggressive cancers.

## 11. Targeting CA IX as a Combinatorial Treatment for Cancer; Future Perspectives

Treating cancer typically requires the use of several therapeutic strategies such as surgery, radiation therapy, and/or chemotherapies [118]. Often therapies must be combined due to efficacy of one preceding the other. For example surgery and radiation therapy, although effective in a vast majority of cases, present limitations in that they can only target confined local regions of neoplastic tissue and are not effective at treating highly metastatic cancer cases [118]. At this stage combinations of multiple chemotherapeutics are usually employed in an attempt to kill cancer cells that have migrated from the primary tumor site. Furthermore, highly aggressive and hypoxic tumors often develop resistance to radiation and certain chemotherapies, or are inoperable; hence alternative or combinations of chemotherapeutics are the only method of treatment available in these particular cases [119,120,121]. This feature of hypoxia and its association with resistance to radiation and chemotherapies has been observed in several cancer types [121,122,123,124,125,126,127]. This is most likely due to several factors including a reduction in overall O_2_ content making the generation of free-radicals needed for radiation therapy extremely difficult, the reduced extracellular pH disrupting functions of alkylating agents, and an upregulation of drug-resistance factors induced by HIFs [1,27]. CA IX, has been linked to cases of therapeutic resistance for several cancers, and is often used as a biomarker for radiation resistance [20,67,120,128,129,130]. As such evidence suggests that a downregulation or inhibition of CA IX allows for an increase in radiation and chemosensitivity in tumors, indicating its potential use as a combinatorial therapy [7,27,118,126,127].

Although the mechanism is not clear, it is proposed that disruption of the differential pH in the tumor miliue and abolishing key regulatory functions of CA IX causes tumor susceptibility to chemo- and radiation-therapies [27]. Despite the unknown mechanism by which CA IX can induce such susceptibility to chemo- or radiation-treatments in once resistance tumors, this observation suggests that CAIs or siRNAs that target CA IX can be used as combinatorial therapies with either radiation or chemotherapy. In addition, recent studies showing that CA IX selective inhibitors can be conjugated to cytotoxic agents suggest that both chemoresistance and tumorigenicity can be combatted using one single agent. This has the potential to not only give patients increased treatment options for aggressive cancer types, but also presents the potential of increasing patient survival rates due to a counter treatment for resistance tumors. Certainly more clinical research is needed to prove that these concepts can be implemented. Overall this presents an exciting way to increase current cancer therapies by use of inhibiting a generic cancer target.

## References

[1] Moulder J.E., Rockwell S. (1987). Tumor hypoxia: Its impact on cancer therapy. Cancer Metastasis Rev..

[2] Peskin B., Carter M.J. (2008). Chronic cellular hypoxia as the prime cause of cancer: What is the de-oxygenating role of adulterated and improper ratios of polyunsaturated fatty acids when incorporated into cell membranes?. Med. Hypotheses.

[3] Vander Heiden M.G., Cantley L.C., Thompson C.B. (2009). Understanding the Warburg Effect: The Metabolic Requirements of Cell Proliferation. Science.

[4] Racker E. (1981). Warburg effect revisited. Science.

[5] Wykoff C.C., Beasley N.J., Watson P.H., Turner K.J., Pastorek J., Sibtain A., Wilson G.D., Turley H., Talks K.L., Maxwell P.H. (2000). Hypoxia-inducible expression of tumor-associated carbonic anhydrases. Cancer Res..

[6] Chia S.K., Wykoff C.C., Watson P.H., Han C., Leek R.D., Pastorek J., Gatter K.C., Ratcliffe P., Harris A.L. (2001). Prognostic significance of a novel hypoxia-regulated marker, carbonic anhydrase IX, in invasive breast carcinoma. J. Clin. Oncol..

[7] McDonald P.C., Winum J.Y., Supuran C.T., Dedhar S. (2012). Recent developments in targeting carbonic anhydrase IX for cancer therapeutics. Oncotarget.

[8] Luo D., Wang Z., Wu J., Jiang C., Wu J. (2014). The Role of Hypoxia Inducible Factor-1 in Hepatocellular Carcinoma. BioMed Res. Int..

[9] Sadri N., Zhang P.J. (2013). Hypoxia-inducible factors: Mediators of cancer progression; prognostic and therapeutic targets in soft tissue sarcomas. Cancers.

[10] Aggarwal M., Boone C.D., Kondeti B., McKenna R. (2013). Structural annotation of human carbonic anhydrases. J. Enzyme Inhib. Med. Chem..

[11] Frost S.C. (2014). Physiological functions of the alpha class of carbonic anhydrases. Subcell. Biochem..

[12] Neri D., Supuran C.T. (2011). Interfering with pH regulation in tumours as a therapeutic strategy. Nat. Rev. Drug Discov..

[13] Li Y., Wang H., Oosterwijk E., Selman Y., Mira J.C., Medrano T., Shiverick K.T., Frost S.C. (2009). Antibody-specific detection of CAIX in breast and prostate cancers. Biochem. Biophys. Res. Commun..

[14] Türeci O., Sahin U., Vollmar E., Siemer S., Göttert E., Seitz G., Parkkila A.K., Shah G.N., Grubb J.H., Pfreundschuh M. (1998). Human carbonic anhydrase XII: cDNA cloning, expression, and chromosomal localization of a carbonic anhydrase gene that is overexpressed in some renal cell cancers. Proc. Natl. Acad. Sci. USA.

[15] Ilie M.I., Hofman V., Ortholan C., Ammadi R.E., Bonnetaud C., Havet K., Venissac N., Mouroux J., Mazure N.M., Pouysségur J. (2011). Overexpression of carbonic anhydrase XII in tissues from resectable non-small cell lung cancers is a biomarker of good prognosis. Int. J. Cancer.

[16] Liao S.Y., Lerman M.I., Stanbridge E.J. (2009). Expression of transmembrane carbonic anhydrases, CAIX and CAXII, in human development. BMC Dev. Biol..

[17] Opavský R., Pastoreková S., Zelník V., Gibadulinová A., Stanbridge E.J., Závada J., Kettmann R., Pastorek J. (1996). Human MN/CA9 gene, a novel member of the carbonic anhydrase family: structure and exon to protein domain relationships. Genomics.

[18] Alterio V., Hilvo M., Di Fiore A., Supuran C.T., Pan P., Parkkila S., Scaloni A., Pastorek J., Pastorekova S., Pedone C. (2009). Crystal structure of the catalytic domain of the tumor-associated human carbonic anhydrase IX. Proc. Natl. Acad. Sci. USA.

[19] De Simone G., Supuran C.T. (2010). Carbonic anhydrase IX: Biochemical and crystallographic characterization of a novel antitumor target. Biochim. Biophys. Acta.

[20] Hilvo M., Baranauskiene L., Salzano A.M., Scaloni A., Matulis D., Innocenti A., Scozzafava A., Monti S.M., Di Fiore A., De Simone G. (2008). Biochemical characterization of CA IX, one of the most active carbonic anhydrase isozymes. J. Biol. Chem..

[21] Pinard M.A., Mahon B.P., McKenna R. (2014). Probing the Surface of Human Carbonic Anhydrase for Clues towards the Design of Isoform Specific Inhibitors. BioMed Res. Int..

[22] Barathova M., Takacova M., Holotnakova T., Gibadulinova A., Ohradanova A., Zatovicova M., Hulikova A., Kopacek J., Parkkila S., Supuran C.T. (2008). Alternative splicing variant of the hypoxia marker carbonic anhydrase IX expressed independently of hypoxia and tumour phenotype. Br. J. Cancer.

[23] Aggarwal M., Kondeti B., McKenna R. (2013). Insights towards sulfonamide drug specificity in α-carbonic anhydrases. Bioorg. Med. Chem..

[24] Kim D.E., Chivian D., Baker D. (2004). Protein structure prediction and analysis using the Robetta server. Nucleic Acids Res..

[25] Pettersen E.F., Goddard T.D., Huang C.C., Couch G.S., Greenblatt D.M., Meng E.C., Ferrin T.E. (2004). UCSF Chimera—a visualization system for exploratory research and analysis. J. Comput. Chem..

[26] Emsley P., Cowtan K. (2004). Coot: Model-building tools for molecular graphics. Acta Crystallogr. D Biol. Crystallogr..

[27] Mahon B.P., McKenna R. (2013). Regulation and role of carbonic anhydrase IX and use as a biomarker and therapeutic target in cancer. Res. Trends Curr. Top. Biochem. Res..

[28] (2013). The PyMOL Molecular Graphics System.

[29] Alterio V., Di Fiore A., D’Ambrosio K., Supuran C.T., de Simone G. (2012). Multiple binding modes of inhibitors to carbonic anhydrases: How to design specific drugs targeting 15 different isoforms?. Chem. Rev..

[30] Supuran C.T. (2013). Carbonic anhydrase inhibitors: an editorial. Expert Opin. Ther. Pat..

[31] Benej M., Pastorekova S., Pastorek J., Frost S.C., McKenna R. (2014). Carbonic Anhydrase IX: Regulation and Role in Cancer. Carbonic Anhydrase: Mechanism, Regulation, Links to Disease, and Industrial Applications.

[32] Wang G.L., Semenza G.L. (1993). General involvement of hypoxia-inducible factor 1 in transcriptional response to hypoxia. Proc. Natl. Acad. Sci. USA.

[33] Wang G.L., Semenza G.L. (1993). Characterization of hypoxia-inducible factor 1 and regulation of DNA binding activity by hypoxia. J. Biol. Chem..

[34] Block K., Gorin Y., Hoover P., Williams P., Chelmicki T., Clark R.A., Yoneda T., Abboud H.E. (2007). NAD(P)H Oxidases Regulate HIF-2 Protein Expression. J. Biol. Chem..

[35] Agarwal E., Brattain M.G., Chowdhury S. (2013). Cell survival and metastasis regulation by AKT signaling in colorectal cancer. Cell. Signal..

[36] Willam C., Masson N., Tian Y.M., Mahmood S.A., Wilson M.I., Bicknell R., Eckardt K.U., Maxwell P.H., Ratcliffe P.J., Pugh C.W. (2002). Peptide blockade of HIFα degradation modulates cellular metabolism and angiogenesis. Proc. Natl. Acad. Sci. USA.

[37] Gallou C., Joly D., Méjean A., Staroz F., Martin N., Tarlet G., Orfanelli M.T., Bouvier R., Droz D., Chrétien Y. (1999). Mutations of the VHL gene in sporadic renal cell carcinoma: Definition of a risk factor for VHL patients to develop an RCC. Hum. Mutat..

[38] Brusselmans K., Bono F., Maxwell P., Dor Y., Dewerchin M., Collen D., Herbert J.M., Carmeliet P. (2001). Hypoxia-inducible factor-2alpha (HIF-2alpha) is involved in the apoptotic response to hypoglycemia but not to hypoxia. J. Biol. Chem..

[39] Sendoel A., Kohler I., Fellmann C., Lowe S.W., Hengartner M.O. (2010). HIF-1 antagonizes p53-mediated apoptosis through a secreted neuronal tyrosinase. Nature.

[40] Wyatt A.W. (2002). Early activation of the p42/p44MAPK pathway mediates adenosine-induced nitric oxide production in human endothelial cells: A novel calcium-insensitive mechanism. FASEB J..

[41] Saarnio J., Parkkila S., Parkkila A.K., Waheed A., Casey M.C., Zhou X.Y., Pastoreková S., Pastorek J., Karttunen T., Haukipuro K. (1998). Immunohistochemistry of carbonic anhydrase isozyme IX (MN/CA IX) in human gut reveals polarized expression in the epithelial cells with the highest proliferative capacity. J. Histochem. Cytochem..

[42] Saarnio J., Parkkila S., Parkkila A.K., Haukipuro K., Pastoreková S., Pastorek J., Kairaluoma M.I., Karttunen T.J. (1998). Immunohistochemical study of colorectal tumors for expression of a novel transmembrane carbonic anhydrase, MN/CA IX, with potential value as a marker of cell proliferation. Am. J. Pathol..

[43] Leibovich B.C., Sheinin Y., Lohse C.M., Thompson R.H., Cheville J.C., Zavada J., Kwon E.D. (2007). Carbonic anhydrase IX is not an independent predictor of outcome for patients with clear cell renal cell carcinoma. J. Clin. Oncol..

[44] Ivanov S., Liao S.Y., Ivanova A., Danilkovitch-Miagkova A., Tarasova N., Weirich G., Merrill M.J., Proescholdt M.A., Oldfield E.H., Lee J. (2001). Expression of hypoxia-inducible cell-surface transmembrane carbonic anhydrases in human cancer. Am. J. Pathol..

[45] Fiaschi T., Giannoni E., Taddei M.L., Cirri P., Marini A., Pintus G., Nativi C., Richichi B., Scozzafava A., Carta F. (2013). Carbonic anhydrase IX from cancer-associated fibroblasts drives epithelial-mesenchymal transition in prostate carcinoma cells. Cell Cycle Georget. Tex.

[46] Eichhorn M. (2013). Mode of action, clinical profile and relevance of carbonic anhydrase inhibitors in glaucoma therapy. Klinische Monatsblätter für Augenheilkunde.

[47] Boone C.D., Pinard M., McKenna R., Silverman D. (2014). Catalytic Mechanism of α-Class Carbonic Anhydrases: CO_2_ Hydration and Proton Transfer. Subcell. Biochem..

[48] Pinard M.A., Boone C.D., Rife B.D., Supuran C.T., McKenna R. (2013). Structural study of interaction between brinzolamide and dorzolamide inhibition of human carbonic anhydrases. Bioorg. Med. Chem..

[49] McKenna R., Supuran C.T. (2014). Carbonic anhydrase inhibitors drug design. Subcell. Biochem..

[50] Kolayli S., Karahalil F., Sahin H., Dincer B., Supuran C.T. (2011). Characterization and inhibition studies of an α-carbonic anhydrase from the endangered sturgeon species Acipenser gueldenstaedti. J. Enzym. Inhib. Med. Chem..

[51] Avvaru B.S., Kim C.U., Sippel K.H., Gruner S.M., Agbandje-McKenna M., Silverman D.N., McKenna R. (2010). A short, strong hydrogen bond in the active site of human carbonic anhydrase II. Biochemistry.

[52] Moeker J., Mahon B.P., Bornaghi L.F., Vullo D., Supuran C.T., McKenna R., Poulsen S.A. (2014). Structural insights into carbonic anhydrase IX isoform specificity of carbohydrate-based sulfamates. J. Med. Chem..

[53] Mahon B.P., Hendon A.M., Driscoll J.M., Rankin G.M., Poulsen S.A., Supuran C.T., McKenna R. (2014). Saccharin: A Lead Compound for Structure-Based Drug Design of Carbonic Anhydrase IX Inhibitors. Bioorg. Med. Chem..

[54] Siebels M., Rohrmann K., Oberneder R., Stahler M., Haseke N., Beck J., Hofmann R., Kindler M., Kloepfer P., Stief C. (2011). A clinical phase I/II trial with the monoclonal antibody cG250 (RENCAREX^®^) and interferon-alpha-2a in metastatic renal cell carcinoma patients. World J. Urol..

[55] Macis G., Di Giovanni S., Di Franco D., Bonomo L. (2013). Future perspectives for diagnostic imaging in urology: from anatomic and functional to molecular imaging. Urologia.

[56] Závada J., Závadová Z., Pastorek J., Biesová Z., Jezek J., Velek J. (2000). Human tumour-associated cell adhesion protein MN/CA IX: identification of M75 epitope and of the region mediating cell adhesion. Br. J. Cancer.

[57] Tomura H., Wang J.Q., Liu J.P., Komachi M., Damirin A., Mogi C., Tobo M., Nochi H., Tamoto K., Im D.S. (2008). Cyclooxygenase-2 expression and prostaglandin E2 production in response to acidic pH through OGR1 in a human osteoblastic cell line. J. Bone Miner. Res..

[58] Supuran C.T. (2008). Carbonic anhydrases: novel therapeutic applications for inhibitors and activators. Nat. Rev. Drug Discov..

[59] Casey J.R., Morgan P.E., Vullo D., Scozzafava A., Mastrolorenzo A., Supuran C.T. (2004). Carbonic anhydrase inhibitors. Design of selective, membrane-impermeant inhibitors targeting the human tumor-associated isozyme IX. J. Med. Chem..

[60] Groves K., Bao B., Zhang J., Handy E., Kennedy P., Cuneo G., Supuran C.T., Yared W., Peterson J.D., Rajopadhye M. (2012). Synthesis and evaluation of near-infrared fluorescent sulfonamide derivatives for imaging of hypoxia-induced carbonic anhydrase IX expression in tumors. Bioorg. Med. Chem. Lett..

[61] Tinker J.P., Coulson R., Weiner I.M. (1981). Dextran-bound inhibitors of carbonic anhydrase. J. Pharmacol. Exp. Ther..

[62] Maren T.H. (1967). Carbonic anhydrase: chemistry, physiology, and inhibition. Physiol. Rev..

[63] Pastorekova S., Casini A., Scozzafava A., Vullo D., Pastorek J., Supuran C.T. (2004). Carbonic anhydrase inhibitors: the first selective, membrane-impermeant inhibitors targeting the tumor-associated isozyme IX. Bioorg. Med. Chem. Lett..

[64] Pastorekova S., Parkkila S., Pastorek J., Supuran C.T. (2004). Carbonic anhydrases: current state of the art, therapeutic applications and future prospects. J. Enzym. Inhib. Med. Chem..

[65] Winum J.Y., Poulsen S.A., Supuran C.T. (2009). Therapeutic applications of glycosidic carbonic anhydrase inhibitors. Med. Res. Rev..

[66] Cousins K.R. (2011). Computer review of ChemDraw Ultra 12.0. J. Am. Chem. Soc..

[67] Moeker J., Peat T.S., Bornaghi L.F., Vullo D., Supuran C.T., Poulsen S.A. (2014). Cyclic secondary sulfonamides: Unusually good inhibitors of cancer-related carbonic anhydrase enzymes. J. Med. Chem..

[68] Lopez M., Bornaghi L.F., Innocenti A., Vullo D., Charman S.A., Supuran C.T., Poulsen S.A. (2010). Sulfonamide Linked Neoglycoconjugates−A New Class of Inhibitors for Cancer-Associated Carbonic Anhydrases. J. Med. Chem..

[69] Meyer H., Vitavska O., Wieczorek H. (2011). Identification of an animal sucrose transporter. J. Cell Sci..

[70] Smith R., Solberg R., Jacobsen L.L., Voreland A.L., Rustan A.C., Thoresen G.H., Johansen H.T. (2014). Simvastatin inhibits glucose metabolism and legumain activity in human myotubes. PLoS One.

[71] Kwon O., Eck P., Chen S., Corpe C.P., Lee J.H., Kruhlak M., Levine M. (2007). Inhibition of the intestinal glucose transporter GLUT2 by flavonoids. FASEB J..

[72] Huttunen K.M., Raunio H., Rautio J. (2011). Prodrugs—From serendipity to rational design. Pharmacol. Rev..

[73] Carroux C.J., Rankin G.M., Moeker J., Bornaghi L.F., Katneni K., Morizzi J., Charman S.A., Vullo D., Supuran C.T., Poulsen S.A. (2013). A prodrug approach toward cancer-related carbonic anhydrase inhibition. J. Med. Chem..

[74] Giang I., Boland E.L., Poon G.M.K. (2014). Prodrug Applications for Targeted Cancer Therapy. AAPS J..

[75] Weidle U.H., Tiefenthaler G., Georges G. (2014). Proteases as activators for cytotoxic prodrugs in antitumor therapy. Cancer Genomics Proteomics.

[76] Moody C.L., Wheelhouse R.T. (2014). The medicinal chemistry of imidazotetrazine prodrugs. Pharm. Basel Switz..

[77] Kehayova P.D., Woodrell C.D., Dostal P.J., Chandra P.P., Jain A. (2002). Phototriggered delivery of hydrophobic carbonic anhydrase inhibitors. Photochem. Photobiol. Sci..

[78] Grandane A., Tanc M., Zalubovskis R., Supuran C.T. (2014). 6-Triazolyl-substituted sulfocoumarins are potent, selective inhibitors of the tumor-associated carbonic anhydrases IX and XII. Bioorg. Med. Chem. Lett..

[79] Woltersdorf O.W., Schwam H., Bicking J.B., Brown S.L., deSolms S.J., Fishman D.R., Graham S.L., Gautheron P.D., Hoffman J.M., Larson R.D. (1989). Topically active carbonic anhydrase inhibitors. 1. O-acyl derivatives of 6-hydroxybenzothiazole-2-sulfonamide. J. Med. Chem..

[80] Hurvitz L.M., Kaufman P.L., Robin A.L., Weinreb R.N., Crawford K., Shaw B. (1991). New developments in the drug treatment of glaucoma. Drugs.

[81] Grove J., Gautheron P., Plazonnet B., Sugrue M.F. (1988). Ocular distribution studies of the topical carbonic anhydrase inhibitors L-643,799 and L-650,719 and related alkyl prodrugs. J. Ocul. Pharmacol..

[82] Barrese A.A., Genis C., Fisher S.Z., Orwenyo J.N., Kumara M.T., Dutta S.K., Phillips E., Kiddle J.J., Tu C., Silverman D.N. (2008). Inhibition of carbonic anhydrase II by thioxolone: A mechanistic and structural study. Biochemistry.

[83] De Simone G., Vitale R.M., Di Fiore A., Pedone C., Scozzafava A., Montero J.L., Winum J.Y., Supuran C.T. (2006). Carbonic anhydrase inhibitors: Hypoxia-activatable sulfonamides incorporating disulfide bonds that target the tumor-associated isoform IX. J. Med. Chem..

[84] Carta F., Maresca A., Scozzafava A., Supuran C.T. (2012). 5- and 6-membered (thio)lactones are prodrug type carbonic anhydrase inhibitors. Bioorg. Med. Chem. Lett..

[85] Reich R., Hoffman A., Veerendhar A., Maresca A., Innocenti A., Supuran C.T., Breuer E. (2012). Carbamoylphosphonates control tumor cell proliferation and dissemination by simultaneously inhibiting carbonic anhydrase IX and matrix metalloproteinase-2. Toward nontoxic chemotherapy targeting tumor microenvironment. J. Med. Chem..

[86] Steiner H., Lindskog S. (1972). Effects of high concentrations of salt on the esterase activity of human carbonic anhydrase. FEBS Lett..

[87] Wong B.C. K., Zhang H., Qin L., Chen H., Fang C., Lu A., Yang Z. (2014). Carbonic anhydrase IX-directed immunoliposomes for targeted drug delivery to human lung cancer cells *in vitro*. Drug Des. Dev. Ther..

[88] Krall N., Pretto F., Decurtins W., Bernardes G.J. L., Supuran C.T., Neri D. (2014). A Small-Molecule Drug Conjugate for the Treatment of Carbonic Anhydrase IX Expressing Tumors. Angew. Chem. Int. Ed..

[89] Shinkai M., Le B., Honda H., Yoshikawa K., Shimizu K., Saga S., Wakabayashi T., Yoshida J., Kobayashi T. (2001). Targeting hyperthermia for renal cell carcinoma using human MN antigen-specific magnetoliposomes. Jpn. J. Cancer Res. Gann.

[90] Torchilin V.P. (2005). Recent advances with liposomes as pharmaceutical carriers. Nat. Rev. Drug Discov..

[91] Krall N., Pretto F., Neri D. (2014). A bivalent small molecule-drug conjugate directed against carbonic anhydrase IX can elicit complete tumour regression in mice. Chem. Sci..

[92] Borel F., Kay M.A., Mueller C. (2014). Recombinant AAV as a platform for translating the therapeutic potential of RNA interference. Mol. Ther. J. Am. Soc. Gene Ther..

[93] Banerjee D., Slack F. (2002). Control of developmental timing by small temporal RNAs: a paradigm for RNA-mediated regulation of gene expression. BioEssays News Rev. Mol. Cell. Dev. Biol..

[94] Ameres S.L., Zamore P.D. (2013). Diversifying microRNA sequence and function. Nat. Rev. Mol. Cell Biol..

[95] Tiram G., Scomparin A., Ofek P., Satchi-Fainaro R. (2014). Interfering cancer with polymeric siRNA nanomedicines. J. Biomed. Nanotechnol..

[96] Sakurai Y., Hatakeyama H., Sato Y., Hyodo M., Akita H., Harashima H. (2013). Gene silencing via RNAi and siRNA quantification in tumor tissue using MEND, a liposomal siRNA delivery system. Mol. Ther. J. Am. Soc. Gene Ther..

[97] Cheng B., Ling C., Dai Y., Lu Y., Glushakova L.G., Gee S.W. Y., McGoogan K.E., Aslanidi G.V., Park M., Stacpoole P.W. (2012). Development of optimized AAV3 serotype vectors: Mechanism of high-efficiency transduction of human liver cancer cells. Gene Ther..

[98] Duan D., Yue Y., Engelhardt J.F. (2001). Expanding AAV packaging capacity with trans-splicing or overlapping vectors: A quantitative comparison. Mol. Ther. J. Am. Soc. Gene Ther..

[99] Ling C., Lu Y., Cheng B., McGoogan K.E., Gee S.W.Y., Ma W., Li B., Aslanidi G.V., Srivastava A. (2011). High-efficiency transduction of liver cancer cells by recombinant adeno-associated virus serotype 3 vectors. J. Vis. Exp..

[100] Weng Y., Fei B., Chi A.L., Cai M. (2013). Inhibition of gastric cancer cell growth *in vivo* by overexpression of adeno-associated virus-mediated survivin mutant C84A. Oncol. Res..

[101] Pan J.G., Luo R.Q., Zhou X., Han R.F., Zeng G.W. (2013). Potent antitumor activity of the combination of HSV-TK and endostatin by adeno-associated virus vector for bladder cancer *in vivo*. Clin. Lab..

[102] Pandya J., Ortiz L., Ling C., Rivers A.E., Aslanidi G. (2014). Rationally designed capsid and transgene cassette of AAV6 vectors for dendritic cell-based cancer immunotherapy. Immunol. Cell Biol..

[103] Pan J.G., Luo R.Q., Zhou X., Han R.F., Zeng G.W. (2013). Suppression of bladder cancer growth by adeno-associated virus vector-mediated combination of HSV-TK and endostatin *in vitro*. Clin. Lab..

[104] Rajendran S., Collins S., van Pijkeren J.P., O’Hanlon D., O’Sullivan G.C., Tangney M. (2011). Targeting of breast metastases using a viral gene vector with tumour-selective transcription. Anticancer Res..

[105] Kozłowska A., Mackiewicz J., Mackiewicz A. (2013). Therapeutic gene modified cell based cancer vaccines. Gene.

[106] Rodriguez-Lebron E., Denovan-Wright E.M., Nash K., Lewin A.S., Mandel R.J. (2005). Intrastriatal rAAV-mediated delivery of anti-huntingtin shRNAs induces partial reversal of disease progression in R6/1 Huntington’s disease transgenic mice. Mol. Ther. J. Am. Soc. Gene Ther..

[107] Ojano-Dirain C., Glushakova L.G., Zhong L., Zolotukhin S., Muzyczka N., Srivastava A., Stacpoole P.W. (2010). An animal model of PDH deficiency using AAV8-siRNA vector-mediated knockdown of pyruvate dehydrogenase E1α. Mol. Genet. Metab..

[108] Gorbatyuk M., Justilien V., Liu J., Hauswirth W.W., Lewin A.S. (2007). Suppression of mouse rhodopsin expression *in vivo* by AAV mediated siRNA delivery. Vis. Res..

[109] Said H.M., Hagemann C., Carta F., Katzer A., Polat B., Staab A., Scozzafava A., Anacker J., Vince G.H., Flentje M. (2013). Hypoxia induced CA9 inhibitory targeting by two different sulfonamide derivatives including acetazolamide in human glioblastoma. Bioorg. Med. Chem..

[110] Lou Y., McDonald P.C., Oloumi A., Chia S., Ostlund C., Ahmadi A., Kyle A., Auf dem Keller U., Leung S., Huntsman D. (2011). Targeting tumor hypoxia: suppression of breast tumor growth and metastasis by novel carbonic anhydrase IX inhibitors. Cancer Res..

[111] Lock F.E., McDonald P.C., Lou Y., Serrano I., Chafe S.C., Ostlund C., Aparicio S., Winum J.Y., Supuran C.T., Dedhar S. (2013). Targeting carbonic anhydrase IX depletes breast cancer stem cells within the hypoxic niche. Oncogene.

[112] Radvak P., Repic M., Svastova E., Takacova M., Csaderova L., Strnad H., Pastorek J., Pastorekova S., Kopacek J. (2013). Suppression of carbonic anhydrase IX leads to aberrant focal adhesion and decreased invasion of tumor cells. Oncol. Rep..

[113] Yu S., Yoon J., Lee J., Myung S., Jang E., Kwak M., Cho E., Jang J., Kim Y., Lee H. (2011). Inhibition of hypoxia-inducible carbonic anhydrase-IX enhances hexokinase II inhibitor-induced hepatocellular carcinoma cell apoptosis. Acta Pharmacol. Sin..

[114] O’Keefe E.P. (2013). siRNAs and shRNAs: Tools for Protein Knockdown by Gene Silencing. Mater. Methods.

[115] Lerch T.F., Xie Q., Chapman M.S. (2010). The structure of adeno-associated virus serotype 3B (AAV-3B): Insights into receptor binding and immune evasion. Virology.

[116] Al-Lazikani B., Banerji U., Workman P. (2012). Combinatorial drug therapy for cancer in the post-genomic era. Nat. Biotechnol..

[117] Meena A.S., Sharma A., Kumari R., Mohammad N., Singh S.V., Bhat M.K. (2013). Inherent and acquired resistance to paclitaxel in hepatocellular carcinoma: Molecular events involved. PLoS One.

[118] Alisi A., Cho W.C., Locatelli F., Fruci D. (2013). Multidrug resistance and cancer stem cells in neuroblastoma and hepatoblastoma. Int. J. Mol. Sci..

[119] Peitzsch C., Perrin R., Hill R.P., Dubrovska A., Kurth I. (2014). Hypoxia as a biomarker for radioresistant cancer stem cells. Int. J. Radiat. Biol..

[120] Sugrue T., Lowndes N.F., Ceredig R. (2014). Hypoxia enhances the radioresistance of mouse mesenchymal stromal cells. Stem Cells Dayt. Ohio.

[121] Wang M., Li X., Qu Y., Xu O., Sun Q. (2013). Hypoxia promotes radioresistance of CD133-positive Hep-2 human laryngeal squamous carcinoma cells *in vitro*. Int. J. Oncol..

[122] Marampon F., Gravina G.L., Zani B.M., Popov V.M., Fratticci A., Cerasani M., Di Genova D., Mancini M., Ciccarelli C., Ficorella C. (2014). Hypoxia sustains glioblastoma radioresistance through ERKs/DNA-PKcs/HIF-1α functional interplay. Int. J. Oncol..

[123] Grosso S., Doyen J., Parks S.K., Bertero T., Paye A., Cardinaud B., Gounon P., Lacas-Gervais S., Noël A., Pouysségur J. (2013). MiR-210 promotes a hypoxic phenotype and increases radioresistance in human lung cancer cell lines. Cell Death Dis..

[124] Crowder S.W., Balikov D.A., Hwang Y.S., Sung H.J. (2014). Cancer Stem Cells under Hypoxia as a Chemoresistance Factor in Breast and Brain. Curr. Pathobiol. Rep..

[125] Cheng Z.X., Wang D.W., Liu T., Liu W.X., Xia W.B., Xu J., Zhang Y.H., Qu Y.K., Guo L.Q., Ding L. (2014). Effects of the HIF-1α and NF-κB loop on epithelial-mesenchymal transition and chemoresistance induced by hypoxia in pancreatic cancer cells. Oncol. Rep..

[126] Vordermark D., Kaffer A., Riedl S., Katzer A., Flentje M. (2005). Characterization of carbonic anhydrase IX (CA IX) as an endogenous marker of chronic hypoxia in live human tumor cells. Int. J. Radiat. Oncol. Biol. Phys..

[127] Ostheimer C., Bache M., Güttler A., Kotzsch M., Vordermark D. (2014). A pilot study on potential plasma hypoxia markers in the radiotherapy of non-small cell lung cancer. Osteopontin, carbonic anhydrase IX and vascular endothelial growth factor. Strahlenther. Onkol..

[128] Brennan D.J., Jirstrom K., Kronblad A., Millikan R.C., Landberg G., Duffy M.J., Rydén L., Gallagher W.M., O’Brien S.L. (2006). CA IX is an independent prognostic marker in premenopausal breast cancer patients with one to three positive lymph nodes and a putative marker of radiation resistance. Clin. Cancer Res..

[129] Anai S., Shiverick K., Medrano T., Nakamura K., Goodison S., Brown B.D., Rosser C.J. (2007). Downregulation of BCL-2 induces downregulation of carbonic anhydrase IX, vascular endothelial growth factor, and pAkt and induces radiation sensitization. Urology.

[130] Dubois L., Peeters S.G.J.A., van Kuijk S.J.A., Yaromina A., Lieuwes N.G., Saraya R., Biemans R., Rami M., Parvathaneni N.K., Vullo D. (2013). Targeting carbonic anhydrase IX by nitroimidazole based sulfamides enhances the therapeutic effect of tumor irradiation: A new concept of dual targeting drugs. Radiother. Oncol..

